# Supplementary figures and images for: Characterizing the limits of human stability during motion: perturbative experiment validates a model-based approach for the Sit-to-Stand task
Source: R Soc Open Sci. 2020 Jan 15;7(1):191410. doi: 10.1098/rsos.191410 (PMC7029948; doi:10.1098/rsos.191410)

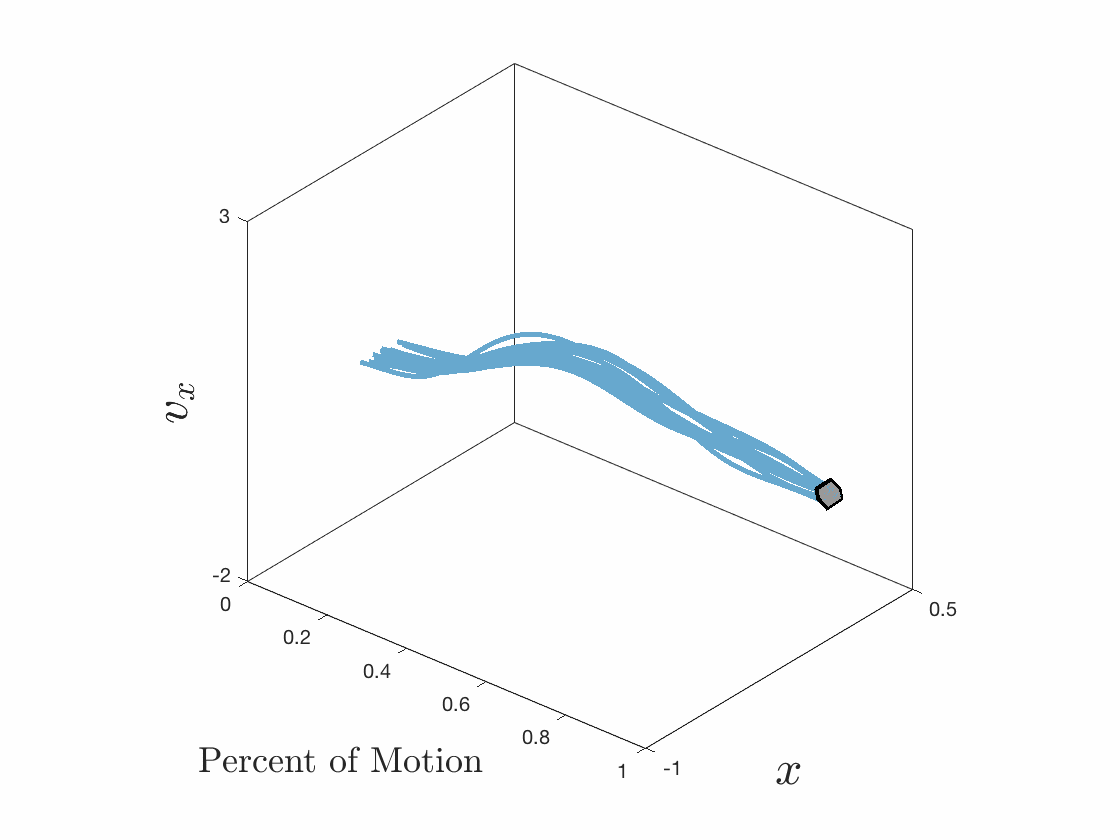

Supplement: Stability Basin creation .gif (horizontal projection) [file rsos191410supp1.gif]

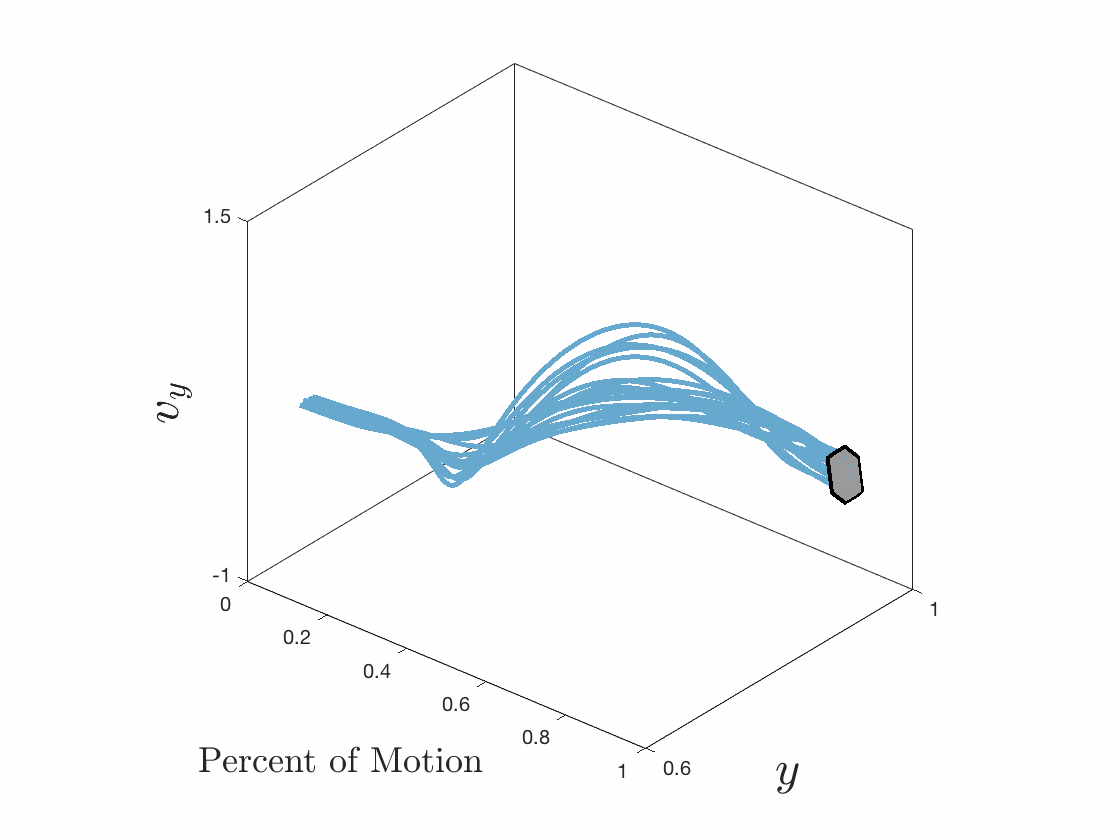

Supplement: Stability Basin creation .gif (vertical projection) [file rsos191410supp2.gif]
