# Supplementary material for: Characterizing the limits of human stability during motion: perturbative experiment validates a model-based approach for the Sit-to-Stand task
Source: R Soc Open Sci. 2020 Jan 15;7(1):191410. doi: 10.1098/rsos.191410 (PMC7029948; doi:10.1098/rsos.191410)
Supplement: Supplementary Tables [file rsos191410supp3.pdf]

Characterizing the limits of human stability during motion: perturbative experiment validates a model-based approach for the Sit-to-Stand task – Supplementary Material to appear in Royal Society Open Science

Patrick D. Holmes[1*], Shannon M. Danforth[1], Xiao-Yu Fu[1,2], Talia Y. Moore[3], and Ram Vasudevan[1,3]

[1]Department of Mechanical Engineering, University of Michigan
[2]Faculty of Kinesiology, University of Calgary
[3]Robotics Institute, University of Michigan
[*]Send correspondence to pdholmes@umich.edu

Supplementary Material Summary

This document includes supplementary results not given in the main text. In particular, the supplementary results present experimental statistics and Stability Basin accuracies for individual subjects and Sit-to-Stand strategies, which are aggregated in the main text. Tables that appear only in the supplementary material are denoted with the prefix “S” before the number.

Table S1. Statistics collected from the perturbative Sit-to-Stand experiment are summarized below. The statistics are given separately for each subject and Sit-to-Stand strategy. Note that the onset of failure (Step %STS and Sit %STS) often occurred after the defined trial end ($t = 1$), which is discussed in Sec. 2.5.1 of the main text.

Subject ID	Strategy	Total	CP	Steps	Sits	Trial Time (s)	Pert. Onset (%STS)	Step Time (%STS)	Sit Time (%STS)
1	MT	29	18	11	1	1.05±0.20	0.38±0.10	0.97±0.28	1.03±0.00
1	N	28	18	7	3	1.38±0.15	0.50±0.07	0.96±0.16	0.97±0.11
1	QS	28	18	2	6	3.24±0.82	0.54±0.04	0.99±0.20	0.75±0.04
2	MT	28	18	1	5	1.18 ± 0.06	0.34 ± 0.04	0.78 ± 0.00	0.79 ± 0.08
2	N	32	18	1	6	1.12 ± 0.11	0.38 ± 0.05	1.64 ± 0.00	0.85 ± 0.13
2	QS	29	18	1	4	1.44 ± 0.41	0.22 ± 0.13	0.66 ± 0.00	0.82 ± 0.29
3	MT	31	18	1	2	1.25 ± 0.20	0.53 ± 0.12	1.55 ± 0.00	0.99 ± 0.01
3	N	28	18	3	0	1.62 ± 0.15	0.45 ± 0.08	1.11 ± 0.42	— ± —
3	QS	30	18	1	2	1.63 ± 0.67	0.35 ± 0.14	1.59 ± 0.00	1.32 ± 0.50
4	MT	27	18	1	3	1.00 ± 0.07	0.45 ± 0.09	1.63 ± 0.00	1.03 ± 0.04
4	N	29	17	1	7	1.36 ± 0.09	0.49 ± 0.06	0.86 ± 0.00	0.90 ± 0.04
4	QS	28	18	4	4	1.87 ± 0.47	0.57 ± 0.12	1.17 ± 0.10	0.83 ± 0.07
5	MT	29	17	3	5	0.92 ± 0.08	0.58 ± 0.07	1.59 ± 0.14	1.17 ± 0.07
5	N	27	18	0	6	1.05 ± 0.06	0.58 ± 0.11	— ± —	1.07 ± 0.05
5	QS	26	18	4	4	2.66 ± 0.47	0.62 ± 0.06	0.97 ± 0.14	0.81 ± 0.06
6	MT	28	18	7	1	1.05 ± 0.06	0.50 ± 0.09	1.11 ± 0.21	1.04 ± 0.00
6	N	29	18	5	5	1.55 ± 0.17	0.52 ± 0.10	1.02 ± 0.15	0.96 ± 0.23
6	QS	26	18	4	4	1.87 ± 0.60	0.55 ± 0.12	0.93 ± 0.07	0.84 ± 0.11
7	MT	28	18	2	1	1.55 ± 0.14	0.42 ± 0.11	1.01 ± 0.02	0.94 ± 0.00
7	N	34	18	7	0	1.00 ± 0.11	0.53 ± 0.10	1.13 ± 0.10	— ± —
7	QS	27	18	1	0	3.09 ± 0.76	0.55 ± 0.12	0.91 ± 0.00	— ± —
8	MT	31	18	1	0	0.97 ± 0.10	0.50 ± 0.10	2.01 ± 0.00	— ± —
8	N	28	18	1	0	1.22 ± 0.16	0.59 ± 0.09	1.43 ± 0.00	— ± —
8	QS	25	17	2	4	2.21 ± 0.48	0.67 ± 0.09	1.14 ± 0.10	0.91 ± 0.05
9	MT	33	18	3	0	1.28 ± 0.20	0.41 ± 0.09	1.21 ± 0.31	— ± —
9	N	31	18	6	0	1.25 ± 0.17	0.49 ± 0.07	1.14 ± 0.17	— ± —
9	QS	27	18	7	1	3.25 ± 0.62	0.62 ± 0.05	0.86 ± 0.09	0.72 ± 0.00
10	MT	30	18	3	0	1.04 ± 0.09	0.49 ± 0.05	1.13 ± 0.37	— ± —
10	N	31	18	6	1	1.12 ± 0.16	0.48 ± 0.08	0.91 ± 0.06	0.84 ± 0.00
10	QS	27	17	7	0	1.35 ± 0.43	0.41 ± 0.14	0.92 ± 0.25	— ± —
11	MT	28	19	7	1	1.18 ± 0.08	0.52 ± 0.06	1.06 ± 0.21	0.99 ± 0.00
11	N	29	18	5	1	1.22 ± 0.13	0.55 ± 0.08	1.11 ± 0.20	0.93 ± 0.00
11	QS	27	18	6	0	2.01 ± 0.42	0.49 ± 0.13	0.81 ± 0.11	— ± —

Table S2. Subject ID 1: The accuracies of the Stability Basins formed using the LQR, FF+FB, and Input Bounds controllers and the Naive method are given below for each of the subject's Sit-to-Stand strategies.

Basin Type	Strategy	Successful Trials	Step Trials	Sit Trials	False Successful Pred.	False Failure Pred.
LQR	MT	2/17 = 11.76%	11/11 = 100.00%	1/1 = 100.00%	0/2 = 0.00%	15/27 = 55.56%
LQR	N	0/18 = 0.00%	7/7 = 100.00%	3/3 = 100.00%	0/0 = —–%	18/28 = 64.29%
LQR	QS	0/20 = 0.00%	2/2 = 100.00%	6/6 = 100.00%	0/0 = —–%	20/28 = 71.43%
LQR	total	2/55 = 3.64%	20/20 = 100.00%	10/10 = 100.00%	0/2 = 0.00%	53/83 = 63.86%
FF+FB	MT	9/17 = 52.94%	11/11 = 100.00%	1/1 = 100.00%	0/9 = 0.00%	8/20 = 40.00%
FF+FB	N	8/18 = 44.44%	7/7 = 100.00%	3/3 = 100.00%	0/8 = 0.00%	10/20 = 50.00%
FF+FB	QS	5/20 = 25.00%	2/2 = 100.00%	6/6 = 100.00%	0/5 = 0.00%	15/23 = 65.22%
FF+FB	total	22/55 = 40.00%	20/20 = 100.00%	10/10 = 100.00%	0/22 = 0.00%	33/63 = 52.38%
Input Bounds	MT	15/17 = 88.24%	11/11 = 100.00%	1/1 = 100.00%	0/15 = 0.00%	2/14 = 14.29%
Input Bounds	N	16/18 = 88.89%	7/7 = 100.00%	3/3 = 100.00%	0/16 = 0.00%	2/12 = 16.67%
Input Bounds	QS	17/20 = 85.00%	2/2 = 100.00%	6/6 = 100.00%	0/17 = 0.00%	3/11 = 27.27%
Input Bounds	total	48/55 = 87.27%	20/20 = 100.00%	10/10 = 100.00%	0/48 = 0.00%	7/37 = 18.92%
Naive	MT	0/17 = 0.00%	11/11 = 100.00%	1/1 = 100.00%	0/0 = —–%	17/29 = 58.62%
Naive	N	2/18 = 11.11%	7/7 = 100.00%	3/3 = 100.00%	0/2 = 0.00%	16/26 = 61.54%
Naive	QS	1/20 = 5.00%	2/2 = 100.00%	6/6 = 100.00%	0/1 = 0.00%	19/27 = 70.37%
Naive	total	3/55 = 5.45%	20/20 = 100.00%	10/10 = 100.00%	0/3 = 0.00%	52/82 = 63.41%

Table S3. Subject ID 2: The accuracies of the Stability Basins formed using the LQR, FF+FB, and Input Bounds controllers and the Naive method are given below for each of the subject's Sit-to-Stand strategies.

Basin Type	Strategy	Successful Trials	Step Trials	Sit Trials	False Successful Pred.	False Failure Pred.
LQR	MT	3/22 = 13.64%	1/1 = 100.00%	5/5 = 100.00%	0/3 = 0.00%	19/25 = 76.00%
LQR	N	0/25 = 0.00%	1/1 = 100.00%	6/6 = 100.00%	0/0 = —–%	25/32 = 78.12%
LQR	QS	4/24 = 16.67%	1/1 = 100.00%	4/4 = 100.00%	0/4 = 0.00%	20/25 = 80.00%
LQR	total	7/71 = 9.86%	3/3 = 100.00%	15/15 = 100.00%	0/7 = 0.00%	64/82 = 78.05%
FF+FB	MT	13/22 = 59.09%	1/1 = 100.00%	5/5 = 100.00%	0/13 = 0.00%	9/15 = 60.00%
FF+FB	N	14/25 = 56.00%	1/1 = 100.00%	6/6 = 100.00%	0/14 = 0.00%	11/18 = 61.11%
FF+FB	QS	3/24 = 12.50%	1/1 = 100.00%	4/4 = 100.00%	0/3 = 0.00%	21/26 = 80.77%
FF+FB	total	30/71 = 42.25%	3/3 = 100.00%	15/15 = 100.00%	0/30 = 0.00%	41/59 = 69.49%
Input Bounds	MT	22/22 = 100.00%	1/1 = 100.00%	5/5 = 100.00%	0/22 = 0.00%	0/6 = 0.00%
Input Bounds	N	25/25 = 100.00%	1/1 = 100.00%	6/6 = 100.00%	0/25 = 0.00%	0/7 = 0.00%
Input Bounds	QS	22/24 = 91.67%	1/1 = 100.00%	4/4 = 100.00%	0/22 = 0.00%	2/7 = 28.57%
Input Bounds	total	69/71 = 97.18%	3/3 = 100.00%	15/15 = 100.00%	0/69 = 0.00%	2/20 = 10.00%
Naive	MT	5/22 = 22.73%	1/1 = 100.00%	5/5 = 100.00%	0/5 = 0.00%	17/23 = 73.91%
Naive	N	3/25 = 12.00%	1/1 = 100.00%	6/6 = 100.00%	0/3 = 0.00%	22/29 = 75.86%
Naive	QS	2/24 = 8.33%	1/1 = 100.00%	4/4 = 100.00%	0/2 = 0.00%	22/27 = 81.48%
Naive	total	10/71 = 14.08%	3/3 = 100.00%	15/15 = 100.00%	0/10 = 0.00%	61/79 = 77.22%

Table S4. Subject ID 3: The accuracies of the Stability Basins formed using the LQR, FF+FB, and Input Bounds controllers and the Naive method are given below for each of the subject's Sit-to-Stand strategies.

Basin Type	Strategy	Successful Trials	Step Trials	Sit Trials	False Successful Pred.	False Failure Pred.
LQR	MT	11/28 = 39.29%	1/1 = 100.00%	2/2 = 100.00%	0/11 = 0.00%	17/20 = 85.00%
LQR	N	0/25 = 0.00%	3/3 = 100.00%	0/0 = —–%	0/0 = —–%	25/28 = 89.29%
LQR	QS	12/27 = 44.44%	1/1 = 100.00%	2/2 = 100.00%	0/12 = 0.00%	15/18 = 83.33%
LQR	total	23/80 = 28.75%	5/5 = 100.00%	4/4 = 100.00%	0/23 = 0.00%	57/66 = 86.36%
FF+FB	MT	19/28 = 67.86%	1/1 = 100.00%	1/2 = 50.00%	1/20 = 5.00%	9/11 = 81.82%
FF+FB	N	13/25 = 52.00%	3/3 = 100.00%	0/0 = —–%	0/13 = 0.00%	12/15 = 80.00%
FF+FB	QS	12/27 = 44.44%	1/1 = 100.00%	2/2 = 100.00%	0/12 = 0.00%	15/18 = 83.33%
FF+FB	total	44/80 = 55.00%	5/5 = 100.00%	3/4 = 75.00%	1/45 = 2.22%	36/44 = 81.82%
Input Bounds	MT	27/28 = 96.43%	1/1 = 100.00%	1/2 = 50.00%	1/28 = 3.57%	1/3 = 33.33%
Input Bounds	N	23/25 = 92.00%	3/3 = 100.00%	0/0 = —–%	0/23 = 0.00%	2/5 = 40.00%
Input Bounds	QS	27/27 = 100.00%	1/1 = 100.00%	1/2 = 50.00%	1/28 = 3.57%	0/2 = 0.00%
Input Bounds	total	77/80 = 96.25%	5/5 = 100.00%	2/4 = 50.00%	2/79 = 2.53%	3/10 = 30.00%
Naive	MT	10/28 = 35.71%	1/1 = 100.00%	1/2 = 50.00%	1/11 = 9.09%	18/20 = 90.00%
Naive	N	5/25 = 20.00%	3/3 = 100.00%	0/0 = —–%	0/5 = 0.00%	20/23 = 86.96%
Naive	QS	3/27 = 11.11%	1/1 = 100.00%	2/2 = 100.00%	0/3 = 0.00%	24/27 = 88.89%
Naive	total	18/80 = 22.50%	5/5 = 100.00%	3/4 = 75.00%	1/19 = 5.26%	62/70 = 88.57%

Table S5. Subject ID 4: The accuracies of the Stability Basins formed using the LQR, FF+FB, and Input Bounds controllers and the Naive method are given below for each of the subject's Sit-to-Stand strategies.

Basin Type	Strategy	Successful Trials	Step Trials	Sit Trials	False Successful Pred.	False Failure Pred.
LQR	MT	2/23 = 8.70%	0/1 = 0.00%	3/3 = 100.00%	1/3 = 33.33%	21/24 = 87.50%
LQR	N	1/21 = 4.76%	1/1 = 100.00%	7/7 = 100.00%	0/1 = 0.00%	20/28 = 71.43%
LQR	QS	6/20 = 30.00%	4/4 = 100.00%	4/4 = 100.00%	0/6 = 0.00%	14/22 = 63.64%
LQR	total	9/64 = 14.06%	5/6 = 83.33%	14/14 = 100.00%	1/10 = 10.00%	55/74 = 74.32%
FF+FB	MT	11/23 = 47.83%	0/1 = 0.00%	3/3 = 100.00%	1/12 = 8.33%	12/15 = 80.00%
FF+FB	N	7/21 = 33.33%	1/1 = 100.00%	7/7 = 100.00%	0/7 = 0.00%	14/22 = 63.64%
FF+FB	QS	9/20 = 45.00%	4/4 = 100.00%	4/4 = 100.00%	0/9 = 0.00%	11/19 = 57.89%
FF+FB	total	27/64 = 42.19%	5/6 = 83.33%	14/14 = 100.00%	1/28 = 3.57%	37/56 = 66.07%
Input Bounds	MT	22/23 = 95.65%	0/1 = 0.00%	3/3 = 100.00%	1/23 = 4.35%	1/4 = 25.00%
Input Bounds	N	21/21 = 100.00%	1/1 = 100.00%	5/7 = 71.43%	2/23 = 8.70%	0/6 = 0.00%
Input Bounds	QS	15/20 = 75.00%	4/4 = 100.00%	4/4 = 100.00%	0/15 = 0.00%	5/13 = 38.46%
Input Bounds	total	58/64 = 90.62%	5/6 = 83.33%	12/14 = 85.71%	3/61 = 4.92%	6/23 = 26.09%
Naive	MT	3/23 = 13.04%	0/1 = 0.00%	3/3 = 100.00%	1/4 = 25.00%	20/23 = 86.96%
Naive	N	1/21 = 4.76%	1/1 = 100.00%	7/7 = 100.00%	0/1 = 0.00%	20/28 = 71.43%
Naive	QS	1/20 = 5.00%	4/4 = 100.00%	4/4 = 100.00%	0/1 = 0.00%	19/27 = 70.37%
Naive	total	5/64 = 7.81%	5/6 = 83.33%	14/14 = 100.00%	1/6 = 16.67%	59/78 = 75.64%

Table S6. Subject ID 5: The accuracies of the Stability Basins formed using the LQR, FF+FB, and Input Bounds controllers and the Naive method are given below for each of the subject's Sit-to-Stand strategies.

Basin Type	Strategy	Successful Trials	Step Trials	Sit Trials	False Successful Pred.	False Failure Pred.
LQR	MT	0/21 = 0.00%	3/3 = 100.00%	5/5 = 100.00%	0/0 = —–%	21/29 = 72.41%
LQR	N	2/21 = 9.52%	0/0 = —–%	6/6 = 100.00%	0/2 = 0.00%	19/25 = 76.00%
LQR	QS	0/18 = 0.00%	4/4 = 100.00%	4/4 = 100.00%	0/0 = —–%	18/26 = 69.23%
LQR	total	2/60 = 3.33%	7/7 = 100.00%	15/15 = 100.00%	0/2 = 0.00%	58/80 = 72.50%
FF+FB	MT	15/21 = 71.43%	3/3 = 100.00%	5/5 = 100.00%	0/15 = 0.00%	6/14 = 42.86%
FF+FB	N	8/21 = 38.10%	0/0 = —–%	6/6 = 100.00%	0/8 = 0.00%	13/19 = 68.42%
FF+FB	QS	5/18 = 27.78%	4/4 = 100.00%	4/4 = 100.00%	0/5 = 0.00%	13/21 = 61.90%
FF+FB	total	28/60 = 46.67%	7/7 = 100.00%	15/15 = 100.00%	0/28 = 0.00%	32/54 = 59.26%
Input Bounds	MT	20/21 = 95.24%	3/3 = 100.00%	4/5 = 80.00%	1/21 = 4.76%	1/8 = 12.50%
Input Bounds	N	21/21 = 100.00%	0/0 = —–%	6/6 = 100.00%	0/21 = 0.00%	0/6 = 0.00%
Input Bounds	QS	16/18 = 88.89%	4/4 = 100.00%	4/4 = 100.00%	0/16 = 0.00%	2/10 = 20.00%
Input Bounds	total	57/60 = 95.00%	7/7 = 100.00%	14/15 = 93.33%	1/58 = 1.72%	3/24 = 12.50%
Naive	MT	3/21 = 14.29%	3/3 = 100.00%	5/5 = 100.00%	0/3 = 0.00%	18/26 = 69.23%
Naive	N	5/21 = 23.81%	0/0 = —–%	6/6 = 100.00%	0/5 = 0.00%	16/22 = 72.73%
Naive	QS	1/18 = 5.56%	4/4 = 100.00%	4/4 = 100.00%	0/1 = 0.00%	17/25 = 68.00%
Naive	total	9/60 = 15.00%	7/7 = 100.00%	15/15 = 100.00%	0/9 = 0.00%	51/73 = 69.86%

Table S7. Subject ID 6: The accuracies of the Stability Basins formed using the LQR, FF+FB, and Input Bounds controllers and the Naive method are given below for each of the subject's Sit-to-Stand strategies.

Basin Type	Strategy	Successful Trials	Step Trials	Sit Trials	False Successful Pred.	False Failure Pred.
LQR	MT	10/20 = 50.00%	7/7 = 100.00%	1/1 = 100.00%	0/10 = 0.00%	10/18 = 55.56%
LQR	N	2/19 = 10.53%	5/5 = 100.00%	5/5 = 100.00%	0/2 = 0.00%	17/27 = 62.96%
LQR	QS	3/18 = 16.67%	4/4 = 100.00%	4/4 = 100.00%	0/3 = 0.00%	15/23 = 65.22%
LQR	total	15/57 = 26.32%	16/16 = 100.00%	10/10 = 100.00%	0/15 = 0.00%	42/68 = 61.76%
FF+FB	MT	15/20 = 75.00%	7/7 = 100.00%	1/1 = 100.00%	0/15 = 0.00%	5/13 = 38.46%
FF+FB	N	6/19 = 31.58%	5/5 = 100.00%	5/5 = 100.00%	0/6 = 0.00%	13/23 = 56.52%
FF+FB	QS	4/18 = 22.22%	4/4 = 100.00%	4/4 = 100.00%	0/4 = 0.00%	14/22 = 63.64%
FF+FB	total	25/57 = 43.86%	16/16 = 100.00%	10/10 = 100.00%	0/25 = 0.00%	32/58 = 55.17%
Input Bounds	MT	19/20 = 95.00%	6/7 = 85.71%	1/1 = 100.00%	1/20 = 5.00%	1/8 = 12.50%
Input Bounds	N	19/19 = 100.00%	5/5 = 100.00%	5/5 = 100.00%	0/19 = 0.00%	0/10 = 0.00%
Input Bounds	QS	16/18 = 88.89%	4/4 = 100.00%	4/4 = 100.00%	0/16 = 0.00%	2/10 = 20.00%
Input Bounds	total	54/57 = 94.74%	15/16 = 93.75%	10/10 = 100.00%	1/55 = 1.82%	3/28 = 10.71%
Naive	MT	4/20 = 20.00%	7/7 = 100.00%	1/1 = 100.00%	0/4 = 0.00%	16/24 = 66.67%
Naive	N	0/19 = 0.00%	5/5 = 100.00%	5/5 = 100.00%	0/0 = ——%	19/29 = 65.52%
Naive	QS	2/18 = 11.11%	4/4 = 100.00%	4/4 = 100.00%	0/2 = 0.00%	16/24 = 66.67%
Naive	total	6/57 = 10.53%	16/16 = 100.00%	10/10 = 100.00%	0/6 = 0.00%	51/77 = 66.23%

Table S8. Subject ID 7: The accuracies of the Stability Basins formed using the LQR, FF+FB, and Input Bounds controllers and the Naive method are given below for each of the subject's Sit-to-Stand strategies.

Basin Type	Strategy	Successful Trials	Step Trials	Sit Trials	False Successful Pred.	False Failure Pred.
LQR	MT	3/25 = 12.00%	2/2 = 100.00%	1/1 = 100.00%	0/3 = 0.00%	22/25 = 88.00%
LQR	N	6/27 = 22.22%	7/7 = 100.00%	0/0 = ——%	0/6 = 0.00%	21/28 = 75.00%
LQR	QS	0/26 = 0.00%	1/1 = 100.00%	0/0 = ——%	0/0 = ——%	26/27 = 96.30%
LQR	total	9/78 = 11.54%	10/10 = 100.00%	1/1 = 100.00%	0/9 = 0.00%	69/80 = 86.25%
FF+FB	MT	13/25 = 52.00%	2/2 = 100.00%	1/1 = 100.00%	0/13 = 0.00%	12/15 = 80.00%
FF+FB	N	12/27 = 44.44%	7/7 = 100.00%	0/0 = ——%	0/12 = 0.00%	15/22 = 68.18%
FF+FB	QS	7/26 = 26.92%	1/1 = 100.00%	0/0 = ——%	0/7 = 0.00%	19/20 = 95.00%
FF+FB	total	32/78 = 41.03%	10/10 = 100.00%	1/1 = 100.00%	0/32 = 0.00%	46/57 = 80.70%
Input Bounds	MT	25/25 = 100.00%	2/2 = 100.00%	1/1 = 100.00%	0/25 = 0.00%	0/3 = 0.00%
Input Bounds	N	27/27 = 100.00%	7/7 = 100.00%	0/0 = ——%	0/27 = 0.00%	0/7 = 0.00%
Input Bounds	QS	25/26 = 96.15%	1/1 = 100.00%	0/0 = ——%	0/25 = 0.00%	1/2 = 50.00%
Input Bounds	total	77/78 = 98.72%	10/10 = 100.00%	1/1 = 100.00%	0/77 = 0.00%	1/12 = 8.33%
Naive	MT	3/25 = 12.00%	2/2 = 100.00%	1/1 = 100.00%	0/3 = 0.00%	22/25 = 88.00%
Naive	N	10/27 = 37.04%	7/7 = 100.00%	0/0 = ——%	0/10 = 0.00%	17/24 = 70.83%
Naive	QS	1/26 = 3.85%	1/1 = 100.00%	0/0 = ——%	0/1 = 0.00%	25/26 = 96.15%
Naive	total	14/78 = 17.95%	10/10 = 100.00%	1/1 = 100.00%	0/14 = 0.00%	64/75 = 85.33%

Table S9. Subject ID 8: The accuracies of the Stability Basins formed using the LQR, FF+FB, and Input Bounds controllers and the Naive method are given below for each of the subject's Sit-to-Stand strategies.

Basin Type	Strategy	Successful Trials	Step Trials	Sit Trials	False Successful Pred.	False Failure Pred.
LQR	MT	2/30 = 6.67%	1/1 = 100.00%	0/0 = —–%	0/2 = 0.00%	28/29 = 96.55%
LQR	N	8/27 = 29.63%	1/1 = 100.00%	0/0 = —–%	0/8 = 0.00%	19/20 = 95.00%
LQR	QS	2/19 = 10.53%	2/2 = 100.00%	4/4 = 100.00%	0/2 = 0.00%	17/23 = 73.91%
LQR	total	12/76 = 15.79%	4/4 = 100.00%	4/4 = 100.00%	0/12 = 0.00%	64/72 = 88.89%
FF+FB	MT	18/30 = 60.00%	1/1 = 100.00%	0/0 = —–%	0/18 = 0.00%	12/13 = 92.31%
FF+FB	N	18/27 = 66.67%	1/1 = 100.00%	0/0 = —–%	0/18 = 0.00%	9/10 = 90.00%
FF+FB	QS	6/19 = 31.58%	2/2 = 100.00%	4/4 = 100.00%	0/6 = 0.00%	13/19 = 68.42%
FF+FB	total	42/76 = 55.26%	4/4 = 100.00%	4/4 = 100.00%	0/42 = 0.00%	34/42 = 80.95%
Input Bounds	MT	30/30 = 100.00%	1/1 = 100.00%	0/0 = —–%	0/30 = 0.00%	0/1 = 0.00%
Input Bounds	N	26/27 = 96.30%	1/1 = 100.00%	0/0 = —–%	0/26 = 0.00%	1/2 = 50.00%
Input Bounds	QS	18/19 = 94.74%	2/2 = 100.00%	4/4 = 100.00%	0/18 = 0.00%	1/7 = 14.29%
Input Bounds	total	74/76 = 97.37%	4/4 = 100.00%	4/4 = 100.00%	0/74 = 0.00%	2/10 = 20.00%
Naive	MT	14/30 = 46.67%	1/1 = 100.00%	0/0 = —–%	0/14 = 0.00%	16/17 = 94.12%
Naive	N	10/27 = 37.04%	1/1 = 100.00%	0/0 = —–%	0/10 = 0.00%	17/18 = 94.44%
Naive	QS	2/19 = 10.53%	2/2 = 100.00%	4/4 = 100.00%	0/2 = 0.00%	17/23 = 73.91%
Naive	total	26/76 = 34.21%	4/4 = 100.00%	4/4 = 100.00%	0/26 = 0.00%	50/58 = 86.21%

Table S10. Subject ID 9: The accuracies of the Stability Basins formed using the LQR, FF+FB, and Input Bounds controllers and the Naive method are given below for each of the subject's Sit-to-Stand strategies.

Basin Type	Strategy	Successful Trials	Step Trials	Sit Trials	False Successful Pred.	False Failure Pred.
LQR	MT	1/30 = 3.33%	3/3 = 100.00%	0/0 = —–%	0/1 = 0.00%	29/32 = 90.62%
LQR	N	1/25 = 4.00%	6/6 = 100.00%	0/0 = —–%	0/1 = 0.00%	24/30 = 80.00%
LQR	QS	0/19 = 0.00%	7/7 = 100.00%	1/1 = 100.00%	0/0 = —–%	19/27 = 70.37%
LQR	total	2/74 = 2.70%	16/16 = 100.00%	1/1 = 100.00%	0/2 = 0.00%	72/89 = 80.90%
FF+FB	MT	19/30 = 63.33%	3/3 = 100.00%	0/0 = —–%	0/19 = 0.00%	11/14 = 78.57%
FF+FB	N	10/25 = 40.00%	6/6 = 100.00%	0/0 = —–%	0/10 = 0.00%	15/21 = 71.43%
FF+FB	QS	0/19 = 0.00%	7/7 = 100.00%	1/1 = 100.00%	0/0 = —–%	19/27 = 70.37%
FF+FB	total	29/74 = 39.19%	16/16 = 100.00%	1/1 = 100.00%	0/29 = 0.00%	45/62 = 72.58%
Input Bounds	MT	30/30 = 100.00%	3/3 = 100.00%	0/0 = —–%	0/30 = 0.00%	0/3 = 0.00%
Input Bounds	N	24/25 = 96.00%	6/6 = 100.00%	0/0 = —–%	0/24 = 0.00%	1/7 = 14.29%
Input Bounds	QS	18/19 = 94.74%	7/7 = 100.00%	0/1 = 0.00%	1/19 = 5.26%	1/8 = 12.50%
Input Bounds	total	72/74 = 97.30%	16/16 = 100.00%	0/1 = 0.00%	1/73 = 1.37%	2/18 = 11.11%
Naive	MT	6/30 = 20.00%	3/3 = 100.00%	0/0 = —–%	0/6 = 0.00%	24/27 = 88.89%
Naive	N	3/25 = 12.00%	6/6 = 100.00%	0/0 = —–%	0/3 = 0.00%	22/28 = 78.57%
Naive	QS	0/19 = 0.00%	7/7 = 100.00%	1/1 = 100.00%	0/0 = —–%	19/27 = 70.37%
Naive	total	9/74 = 12.16%	16/16 = 100.00%	1/1 = 100.00%	0/9 = 0.00%	65/82 = 79.27%

Table S11. Subject ID 10: The accuracies of the Stability Basins formed using the LQR, FF+FB, and Input Bounds controllers and the Naive method are given below for each of the subject's Sit-to-Stand strategies.

Basin Type	Strategy	Successful Trials	Step Trials	Sit Trials	False Successful Pred.	False Failure Pred.
LQR	MT	1/27 = 3.70%	3/3 = 100.00%	0/0 = —–%	0/1 = 0.00%	26/29 = 89.66%
LQR	N	1/24 = 4.17%	6/6 = 100.00%	1/1 = 100.00%	0/1 = 0.00%	23/30 = 76.67%
LQR	QS	6/20 = 30.00%	7/7 = 100.00%	0/0 = —–%	0/6 = 0.00%	14/21 = 66.67%
LQR	total	8/71 = 11.27%	16/16 = 100.00%	1/1 = 100.00%	0/8 = 0.00%	63/80 = 78.75%
FF+FB	MT	20/27 = 74.07%	3/3 = 100.00%	0/0 = —–%	0/20 = 0.00%	7/10 = 70.00%
FF+FB	N	15/24 = 62.50%	6/6 = 100.00%	1/1 = 100.00%	0/15 = 0.00%	9/16 = 56.25%
FF+FB	QS	7/20 = 35.00%	7/7 = 100.00%	0/0 = —–%	0/7 = 0.00%	13/20 = 65.00%
FF+FB	total	42/71 = 59.15%	16/16 = 100.00%	1/1 = 100.00%	0/42 = 0.00%	29/46 = 63.04%
Input Bounds	MT	27/27 = 100.00%	1/3 = 33.33%	0/0 = —–%	2/29 = 6.90%	0/1 = 0.00%
Input Bounds	N	24/24 = 100.00%	6/6 = 100.00%	1/1 = 100.00%	0/24 = 0.00%	0/7 = 0.00%
Input Bounds	QS	19/20 = 95.00%	4/7 = 57.14%	0/0 = —–%	3/22 = 13.64%	1/5 = 20.00%
Input Bounds	total	70/71 = 98.59%	11/16 = 68.75%	1/1 = 100.00%	5/75 = 6.67%	1/13 = 7.69%
Naive	MT	6/27 = 22.22%	3/3 = 100.00%	0/0 = —–%	0/6 = 0.00%	21/24 = 87.50%
Naive	N	4/24 = 16.67%	6/6 = 100.00%	1/1 = 100.00%	0/4 = 0.00%	20/27 = 74.07%
Naive	QS	3/20 = 15.00%	7/7 = 100.00%	0/0 = —–%	0/3 = 0.00%	17/24 = 70.83%
Naive	total	13/71 = 18.31%	16/16 = 100.00%	1/1 = 100.00%	0/13 = 0.00%	58/75 = 77.33%

Table S12. Subject ID 11: The accuracies of the Stability Basins formed using the LQR, FF+FB, and Input Bounds controllers and the Naive method are given below for each of the subject's Sit-to-Stand strategies.

Basin Type	Strategy	Successful Trials	Step Trials	Sit Trials	False Successful Pred.	False Failure Pred.
LQR	MT	0/20 = 0.00%	7/7 = 100.00%	1/1 = 100.00%	0/0 = —–%	20/28 = 71.43%
LQR	N	2/23 = 8.70%	5/5 = 100.00%	1/1 = 100.00%	0/2 = 0.00%	21/27 = 77.78%
LQR	QS	1/21 = 4.76%	6/6 = 100.00%	0/0 = —–%	0/1 = 0.00%	20/26 = 76.92%
LQR	total	3/64 = 4.69%	18/18 = 100.00%	2/2 = 100.00%	0/3 = 0.00%	61/81 = 75.31%
FF+FB	MT	7/20 = 35.00%	7/7 = 100.00%	1/1 = 100.00%	0/7 = 0.00%	13/21 = 61.90%
FF+FB	N	14/23 = 60.87%	4/5 = 80.00%	1/1 = 100.00%	1/15 = 6.67%	9/14 = 64.29%
FF+FB	QS	9/21 = 42.86%	6/6 = 100.00%	0/0 = —–%	0/9 = 0.00%	12/18 = 66.67%
FF+FB	total	30/64 = 46.88%	17/18 = 94.44%	2/2 = 100.00%	1/31 = 3.23%	34/53 = 64.15%
Input Bounds	MT	20/20 = 100.00%	4/7 = 57.14%	1/1 = 100.00%	3/23 = 13.04%	0/5 = 0.00%
Input Bounds	N	22/23 = 95.65%	4/5 = 80.00%	1/1 = 100.00%	1/23 = 4.35%	1/6 = 16.67%
Input Bounds	QS	20/21 = 95.24%	6/6 = 100.00%	0/0 = —–%	0/20 = 0.00%	1/7 = 14.29%
Input Bounds	total	62/64 = 96.88%	14/18 = 77.78%	2/2 = 100.00%	4/66 = 6.06%	2/18 = 11.11%
Naive	MT	2/20 = 10.00%	7/7 = 100.00%	1/1 = 100.00%	0/2 = 0.00%	18/26 = 69.23%
Naive	N	6/23 = 26.09%	4/5 = 80.00%	1/1 = 100.00%	1/7 = 14.29%	17/22 = 77.27%
Naive	QS	4/21 = 19.05%	6/6 = 100.00%	0/0 = —–%	0/4 = 0.00%	17/23 = 73.91%
Naive	total	12/64 = 18.75%	17/18 = 94.44%	2/2 = 100.00%	1/13 = 7.69%	52/71 = 73.24%
